# Author Correction: Engineering Auger recombination in colloidal quantum dots via dielectric screening

**DOI:** 10.1038/s41467-019-11159-z

**Published:** 2019-07-09

**Authors:** Xiaoqi Hou, Jun Kang, Haiyan Qin, Xuewen Chen, Junliang Ma, Jianhai Zhou, Liping Chen, Linjun Wang, Lin-Wang Wang, Xiaogang Peng

**Affiliations:** 1 Center for Chemistry of Novel & High-Performance Materials, and Department of Chemistry, Zhejiang University, 310027 Hangzhou, People’s Republic of China; 2 Material Science Division, Lawrence Berkeley National Laboratory, Berkeley, CA 94720 USA; 3 School of Physics, Huazhong University of Science and Technology, 430074 Wuhan, People’s Republic of China

**Keywords:** Optical materials, Quantum dots

Correction to: *Nature Communications* 10.1038/s41467-019-09737-2, published online 15 April 2019.

The original version of this Article contained an error in the Acknowledgements, which incorrectly omitted from the end the following: ‘J. Kang and L.W. Wang were supported by the Director, Office of Science, the Office of Basic Energy Sciences (BES), Materials Sciences and Engineering (MSE) Division of the U.S. Department of Energy (DOE) through the organic/inorganic nanocomposite program (KC3104) under contract DE-AC02-05CH11231. It used the computational resource of the Oak Ridge Leadership Computing Facility at the Oak Ridge National Laboratory through the INCITE project.’ This has been corrected in both the PDF and HTML versions of the Article.

