# Supplementary material for: Engineering Auger recombination in colloidal quantum dots via dielectric screening
Source: Nat Commun. 2019 Apr 15;10:1750. doi: 10.1038/s41467-019-09737-2 (PMC6465357; doi:10.1038/s41467-019-09737-2)
Supplement: Supplementary file 1 — Supplementary Information [file 41467_2019_9737_MOESM1_ESM.pdf]

Supplementary Information

Engineering Auger recombination in colloidal quantum dots via dielectric screening

Xiaoqi Hou[1], Jun Kang[2], Haiyan Qin[1]*, Xuewen Chen[3], Junliang Ma[1], Jianhai Zhou[1], Liping Chen[1], Linjun Wang[1], Lin-Wang Wang[2]*, and Xiaogang Peng[1]*

[1]*Center for Chemistry of Novel & High-Performance Materials, and Department of Chemistry, Zhejiang University, Hangzhou, 310027, P. R. China*

[2]*Material Science Division, Lawrence Berkeley National Laboratory, Berkeley, California 94720, USA.*

[3]*School of Physics, Huazhong University of Science and Technology, Wuhan 430074, P. R. China*

Corresponding to hattieqin@zju.edu.cn, lwwang@lbl.gov and xpeng@zju.edu.cn.

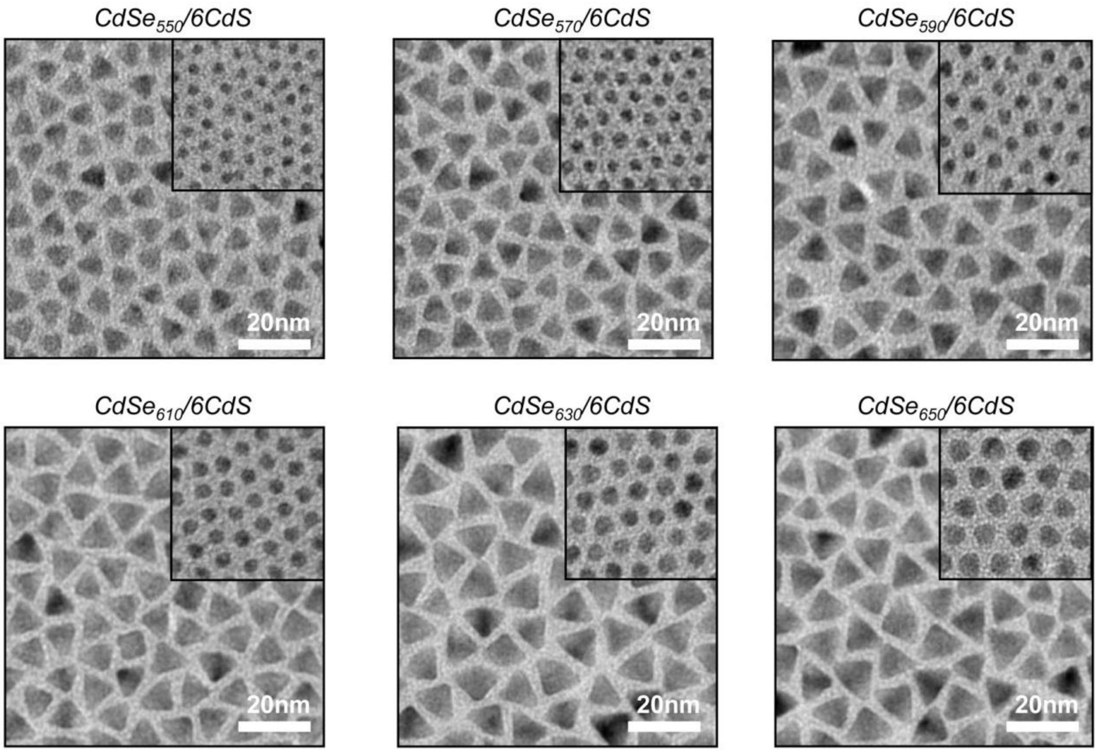

Supplementary Figure 1. TEM images of core/shell QDs with different CdSe core sizes and 6 monolayers of CdS shell. Insets: TEM images of the corresponding CdSe core.

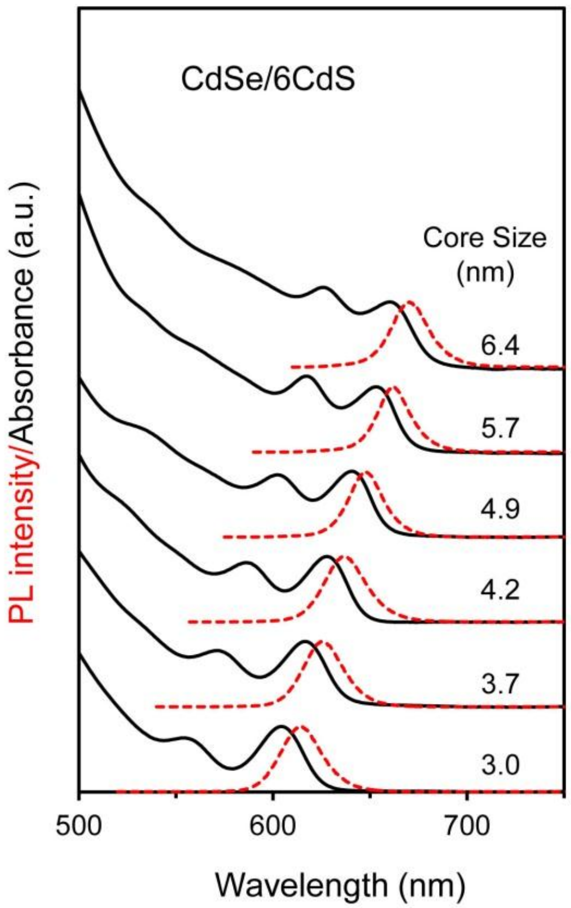

Supplementary Figure 2. Absorption and PL spectra of CdSe/CdS core/shell QDs with different core sizes and 6 monolayers of shell.

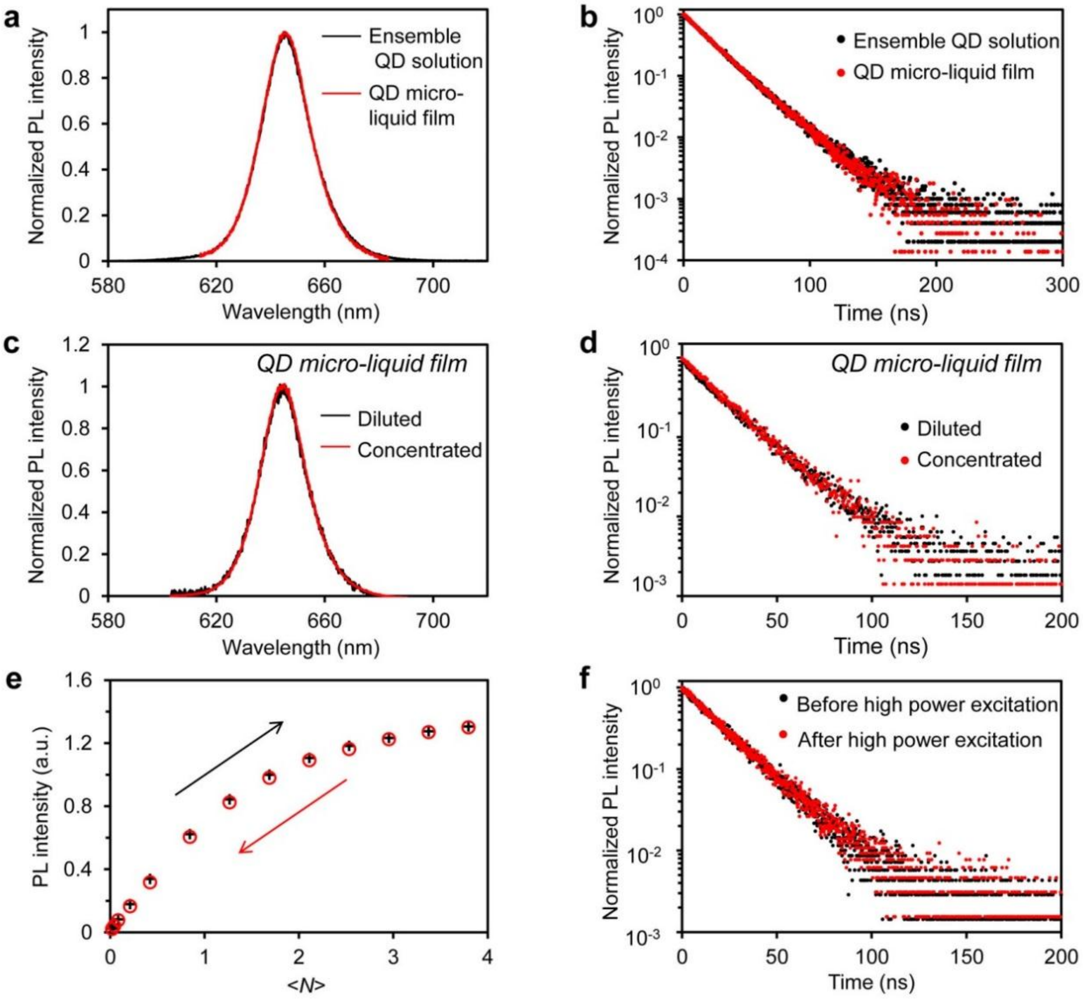

Supplementary Figure 3. Optical properties of QD micro-liquid film. **a,** Normalized PL spectra and **b,** PL decay curves of ensemble QD solution (black) and corresponding QD micro-liquid film (red). **c,** Normalized PL spectra and **d,** PL decay curves of QD micro-liquid films with different concentrations. ~6 QD/μm^3 for diluted QD solution (black) and ~60 QD/μm^3 for concentrated QD solution (red). **e,** PL saturation for QD micro-liquid film measured with increasing (black cross) and decreasing (red circle) excitation power. **f,** PL decay curves of QD micro-liquid film under low power excitation before (black) and after high power excitation (red).

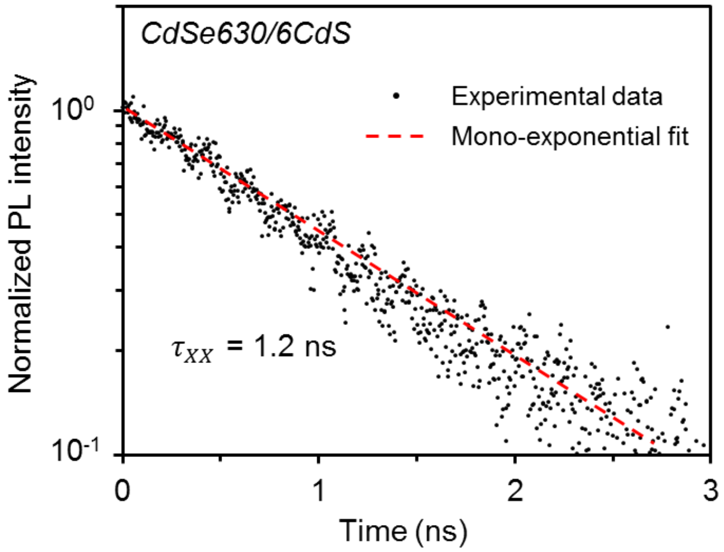

Supplementary Figure 4. Biexciton decay dynamics for CdSe/CdS core/shell QDs with the first-exciton absorption peak of core at 630 nm and 6 monolayers of shell. The biexciton decay dynamics is extracted by subtraction of the two PL decay curves in Fig. 1e. The red dashed line is the mono-exponential fit of the extracted decay curve.

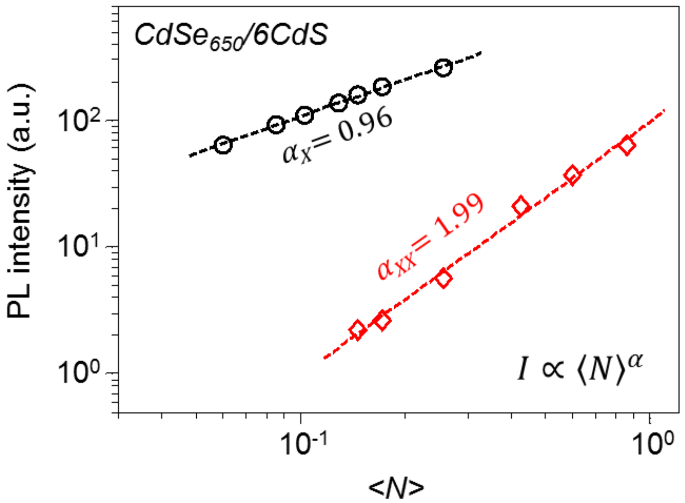

Supplementary Figure 5. Excitation-power dependence of emission intensity of single-exciton (black circles) and biexciton (red diamonds) for CdSe/CdS core/shell QDs with the first-exciton absorption peak of core at 650 nm and 6 monolayers of shell. The slopes in the log-log plot for both channels match the expected values.

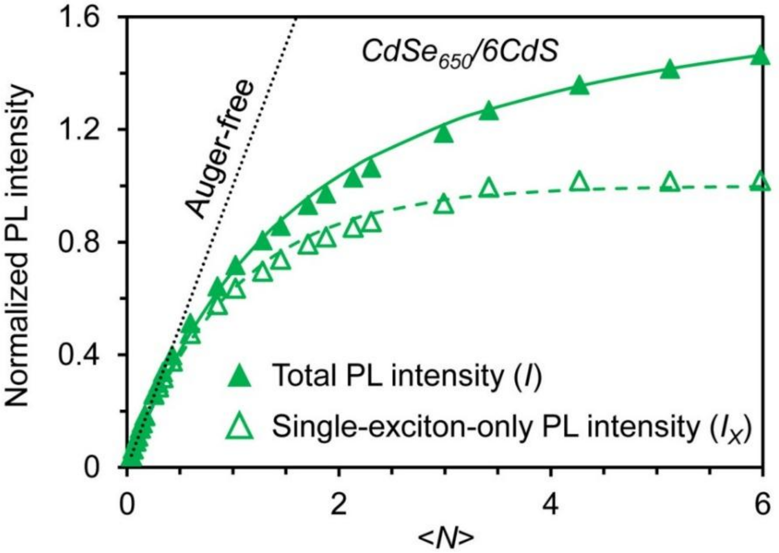

Supplementary Figure 6. PL saturation along with excitation power increase for $CdSe_{650}$/6CdS micro-liquid film. Curves of total PL intensity (I) and the single-exciton-only PL intensity fraction (I_X) versus $\langle N \rangle$ are fitted by a saturation function with biexciton QY as a fitting parameter (see below for details). For each excitation power, PL decay curve is measured in a fixed period of time. The single-exciton-only PL intensity (I_X) is proportional to the total photon number of the long lifetime (single-exciton) component of the PL decay curve.

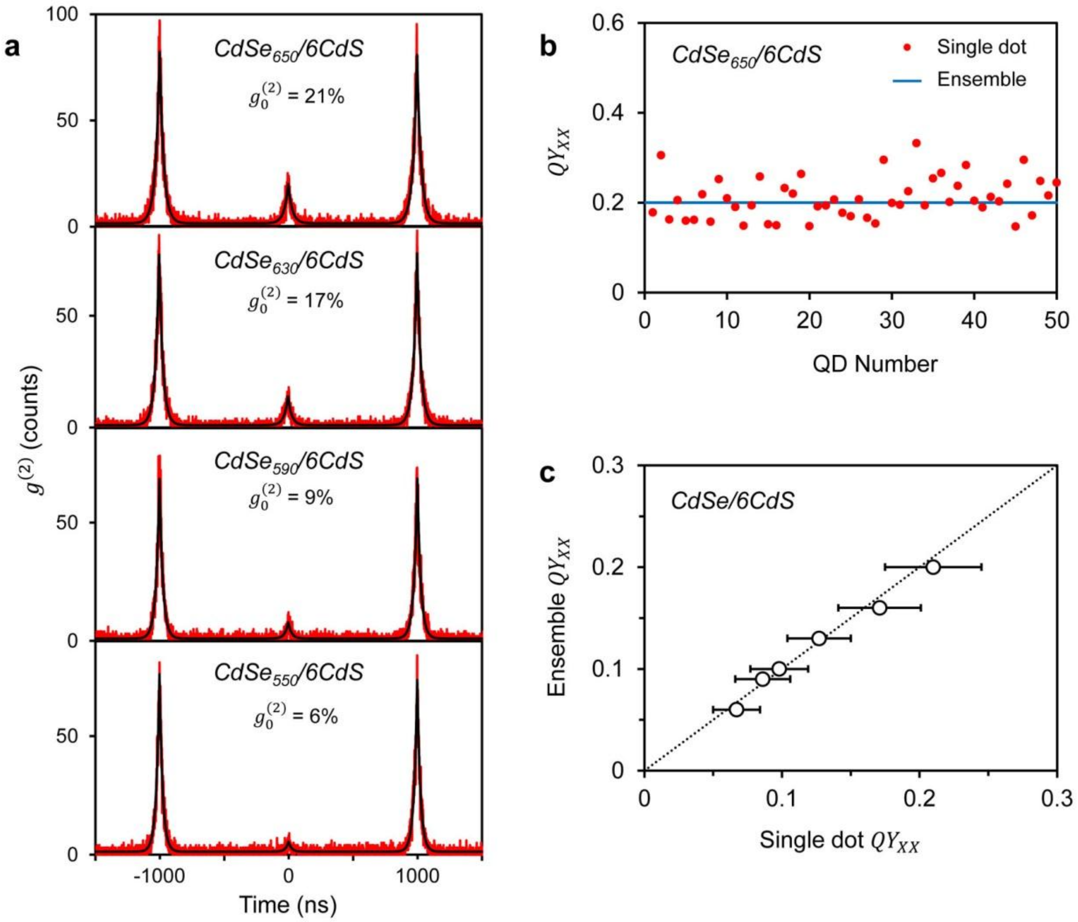

Supplementary Figure 7. Biexicton PL QY of CdSe/CdS core/shell QDs measured in single-dot and ensemble levels. **a,** Representative $g^{(2)}$ traces (red) with fittings (black) of single CdSe/CdS QDs with different core sizes and 6 monolayers of shell. $g_0^{(2)}$ is the biexciton QY while single exciton QY is close to unity. **b,** Biexciton QYs of 50 single CdSe/CdS QDs with the first-exciton absorption peak of core at 650 nm and 6 monolayers of shell measured via second-order photon intensity correlation measurement (red circle) and ensemble biexciton QYs measured via PL saturation experiment with QD micro-liquid film (blue line). **c,** Ensemble biexciton QYs measured using QD micro-liquid film versus the single-dot values measured via second-order photon intensity correlation measurements for CdSe/CdS QDs with different core sizes and 6 monolayers of shell. The error bar is the standard deviation for 50 single QDs for each sample. The results show a great consistency of ensemble biexciton QYs measured using QD micro-liquid film and the single dot values measured via second-order photon intensity correlation measurements.

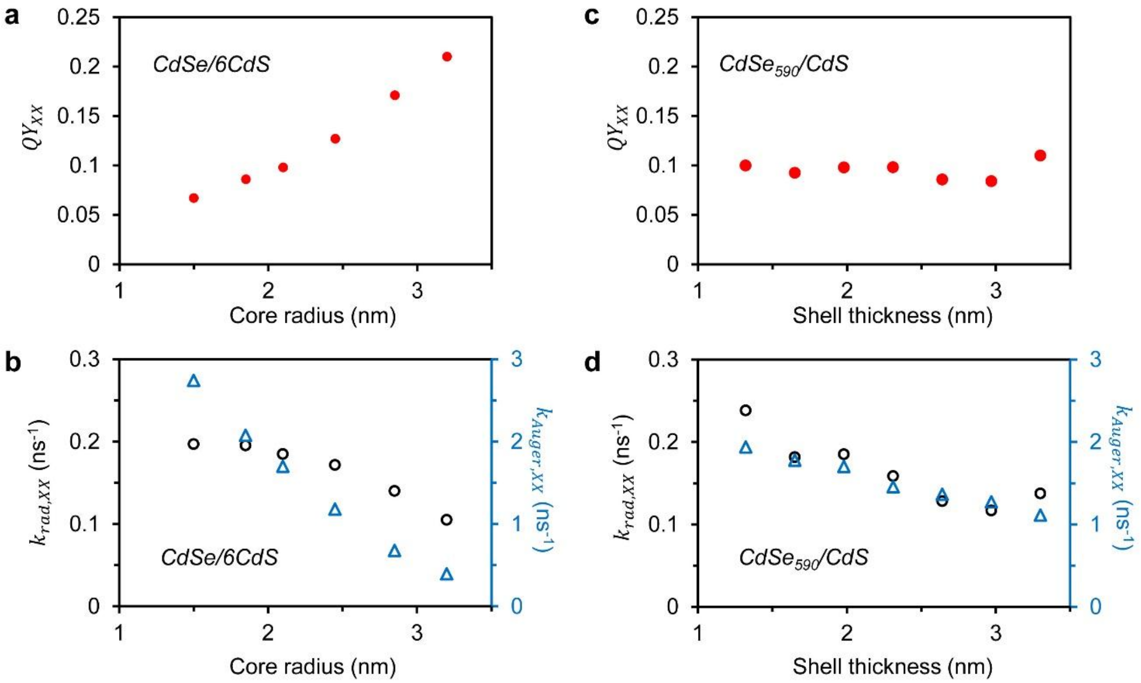

Supplementary Figure 8. Core radius and shell thickness dependent biexciton properties. **a,** Biexciton quantum yield and **b,** radiative recombination rates and nonradiative Auger recombination rates for CdSe/CdS core/shell QDs with different core radiuses and 6 monolayers of CdS shell. **c,** Biexciton quantum yield and **d,** radiative recombination rates and nonradiative Auger recombination rates for CdSe/CdS core/shell QDs with 2.1 nm core radius (the first abs peak for core at 590 nm) and different monolayers of CdS shell.

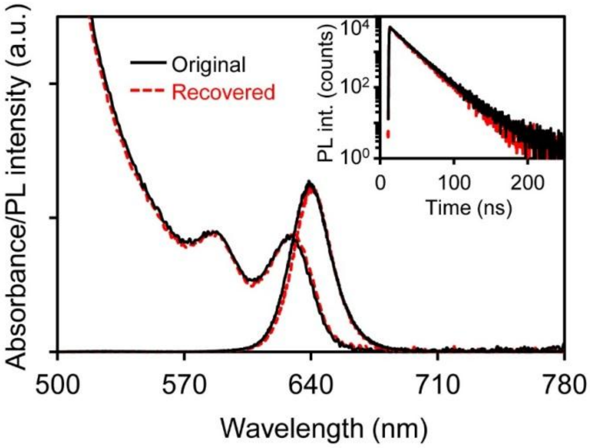

Supplementary Figure 9. Recoverability of photochemical electron-doping of CdSe/CdS core/shell QDs. PL spectra, absorption spectra and PL decay curves of $CdSe_{590}$/8CdS before photochemical doping (black) and after re-oxidation by exposure to air (red).The PL spectrum, absorption spectrum and PL decay dynamics of the recovered QDs are identical to the original ones. This indicated that the photochemical electron doping experiment is a reversible process that not harmful to the structure or surface passivation of the QDs.

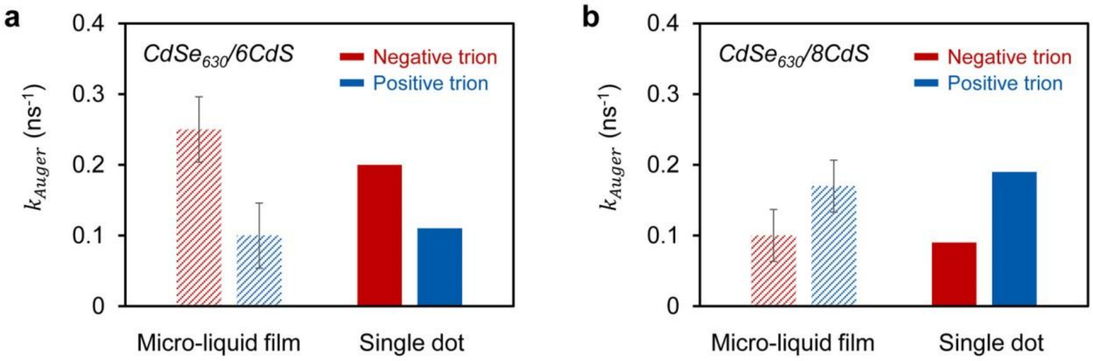

Supplementary Figure 10. Auger rate of positive trion calculated via different methods. **a,** The Auger recombination rates of negative and positive trions for CdSe/CdS core/shell QDs with the first-exciton absorption peak at 630 nm and 6 monolayers of shell. The $k_{Auger,X^+}$is calculated with $k_{Auger,XX}$ measured by QD micro-liquid film (hatched bar) or measured using single-dot photon statistics experiment for the same single dot (solid bar). Error bars are defined as s.d. **b,** The same for CdSe/CdS core/shell QDs with the first-exciton absorption peak at 630 nm and 8 monolayers of shell.

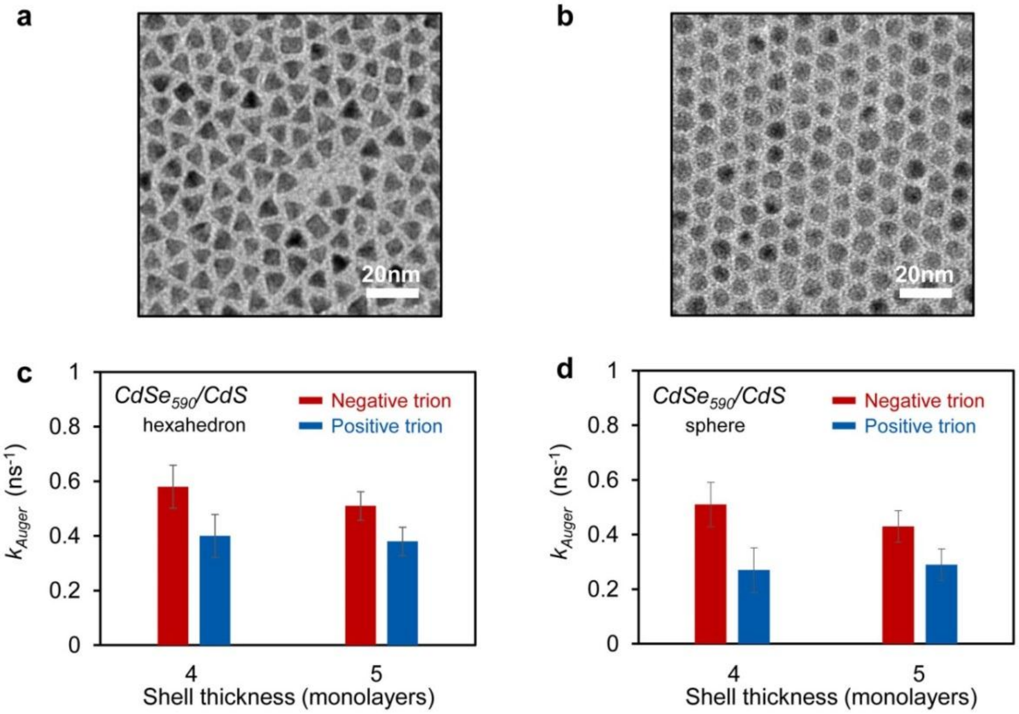

Supplementary Figure 11. Auger rates of negative and positive trions for CdSe/CdS core/shell QDs with hexahedral and spherical shape. TEM images of **a,** hexahedral and **b,** spherical CdSe/CdS core/shell QDs with the first-exciton absorption peak of core at 590 nm and 5 monolayers of shell. The spherical QDs were converted from the hexahedral counterpart. **c, d,** Auger recombination rates for negative and positive trions for hexahedral and spherical CdSe/CdS core/shell QDs with the first-exciton absorption peak of core at 590 nm and different shell thicknesses. Error bars are defined as s.d. These results suggest that the shape of QDs has limit influence on the Auger recombination rates.

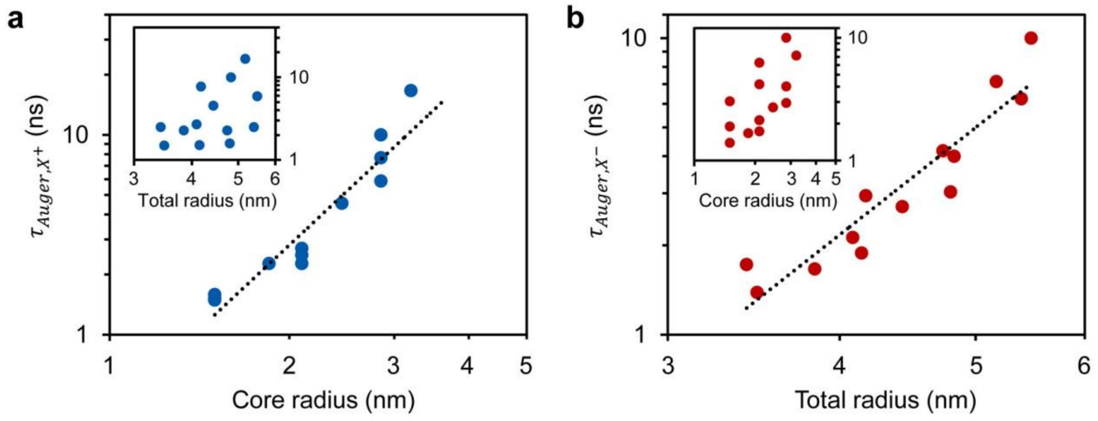

Supplementary Figure 12. Charge-dependent volume scaling of Auger recombination. **a,** Log-log plot for Auger recombination lifetime of positive trion (τ_{Auger,X^+}) versus core radius and total radius (inset). **b,** Log-log plot for Auger recombination lifetime of negative trion (τ_{Auger,X^-}) versus total radius and core radius (inset). Dashed lines are power-law fits with exponents of 2.8 and 3.7 for τ_{Auger,X^+} to core radius and τ_{Auger,X^-} to total radius respectively.

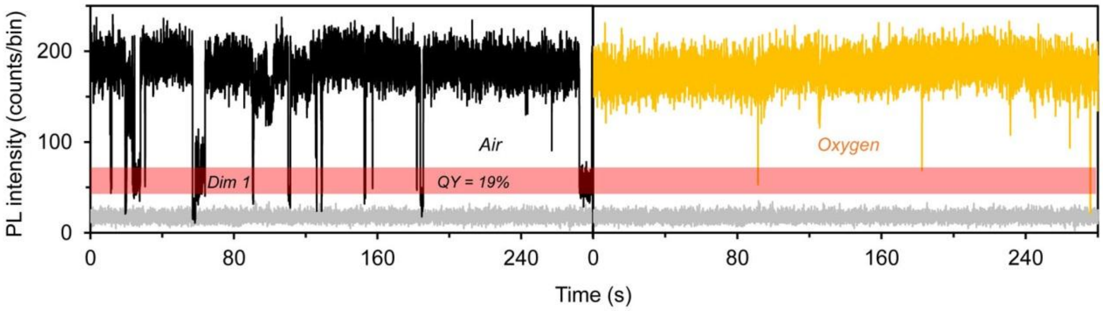

Supplementary Figure 13. Atmosphere dependent experiment. Representative PL intensity trajectories of a single CdSe/CdS core/shell QDs with the first exciton absorption peak of core at 630 nm and 4 monolayers of shell during changing the surrounding atmosphere from air (black curve) to oxygen (yellow curve).

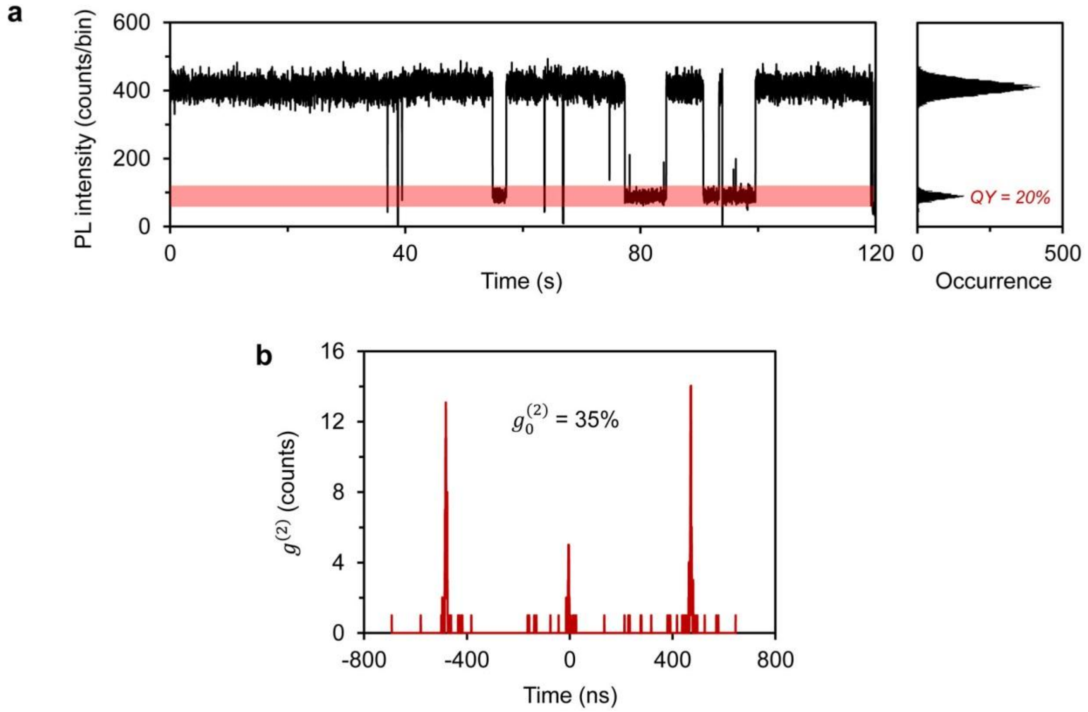

Supplementary Figure 14. A second-order photon intensity correlation measurement for the dim state. **a,** A PL intensity trajectory of a single CdSe/CdS core/shell QDs with the first exciton absorption peak of core at 630 nm and 4 monolayers of shell under low power excitation ($<N> \approx 0.05$) and the corresponding histogram. Bin time is 10 ms. **b,** the $g^{(2)}$ trace of the dim state (indicated as red background in **a**). The repetition frequency of the excitation was 2.1 MHz.

Supplementary Table 1. PL properties of ensemble CdSe/CdS core/shell QDs with 2 nm shell thickness but different core sizes. The PL lifetime τ_X is obtained by fitting the PL decay curves with a single-exponential decay function. χ_R^2 is the goodness-of-fit.

QD sample	Core size (nm)	Shell thickness (nm)	PL peak position (nm)	PL FWHM (meV)	PL lifetime τ_X (ns)	χ_R^2	PL QY (%)
$CdSe_{550}/6CdS$	3.0	2.0	613	80	20.2	1.25	95
$CdSe_{570}/6CdS$	3.7	2.0	625	75	20.5	1.06	97
$CdSe_{590}/6CdS$	4.2	2.0	636	67	21.0	1.10	96
$CdSe_{610}/6CdS$	4.9	2.0	648	61	21.5	1.01	96
$CdSe_{630}/6CdS$	5.7	2.0	661	55	22.3	1.07	94
$CdSe_{650}/6CdS$	6.4	2.0	670	61	22.7	1.13	93

Supplementary Table 2. PL properties of ensemble CdSe/CdS core/shell QDs with 4.2 nm core size and different shell thicknesses. The PL lifetime τ_X is obtained by fitting the PL decay curves with a single-exponential decay function. χ_R^2 is the goodness-of-fit.

QD sample	Shell thickness (nm)	Shell monolayers	PL peak position (nm)	PL FWHM (meV)	PL lifetime τ_X (ns)	χ_R^2	PL QY (%)
$CdSe_{590}/4CdS$	1.3	4	627	70	18.5	1.09	94
$CdSe_{590}/5CdS$	1.6	5	632	68	19.3	1.17	92
$CdSe_{590}/6CdS$	2.0	6	636	67	21.0	1.10	96
$CdSe_{590}/7CdS$	2.3	7	639	75	21.5	1.04	98
$CdSe_{590}/8CdS$	2.6	8	641	68	23.0	1.01	97
$CdSe_{590}/9CdS$	3.0	9	643	66	24.0	1.22	96
$CdSe_{590}/10CdS$	3.3	10	645	70	24.8	1.27	95

Supplementary Table 3. Radiative rate of neural single exciton, and emission rate, emission quantum yield, radiative rate and Auger non-radiative rate of negative trion for CdSe/CdS QDs with different core sizes or shell thicknesses. The data is determined with single dot spectroscopy.

QD sample	k_X (ns^{-1})	k_{X^-} (ns^{-1})	QY_{X^-} (%)	$k_{rad\ X^-}$ (ns^{-})	$k_{Auger\ X^-}$(ns^{-})
$CdSe_{590}$/4CdS	0.045 $\pm$ 0.003	0.64 $\pm$ 0.11	10 $\pm$ 2.5	0.067 $\pm$ 0.02	0.58 $\pm$ 0.09
$CdSe_{590}$/6CdS	0.042 $\pm$ 0.005	0.53 $\pm$ 0.07	12 $\pm$ 1.0	0.063 $\pm$ 0.01	0.47 $\pm$ 0.06
$CdSe_{590}$/8CdS	0.038 $\pm$ 0.003	0.30 $\pm$ 0.03	20 $\pm$ 0.7	0.061 $\pm$ 0.005	0.24 $\pm$ 0.02
$CdSe_{630}$/6CdS	0.039 $\pm$ 0.004	0.31 $\pm$ 0.05	24 $\pm$ 4.7	0.073 $\pm$ 0.006	0.25 $\pm$ 0.05

Supplementary Table 4. PL quantum yields (QYs), lifetimes, radiative and nonradiative Auger recombination rates of single-exciton, negative and positive trion states for CdSe/CdS core/shell QDs with the first exciton absorption peak of core at 630 nm and 8 monolayers of shell.

$CdSe_{630}$/8CdS	QY (%)	Total lifetime, τ_{tot} (ns)	Radiative rate, k_{rad} (ns^{-1})	Auger rate, k_{Auger} (ns^{-1})
X	100	27	0.037	NA
X^-	46	6.7	0.069	0.081
X^+	31	4.3	0.072	0.16

Supplementary Table 5. PL quantum yields (QYs), lifetimes, radiative and nonradiative Auger recombination rates of single-exciton, negative and positive trion states for CdSe/CdS core/shell QDs with the first exciton absorption peak of core at 630 nm and 4 monolayers of shell.

$CdSe_{630}$/4CdS	QY (%)	Total lifetime τ_{tot} (ns)	Radiative rate k_{rad} (ns^{-1})	Auger rate k_{Auger} (ns^{-1})
X	100	22.7	0.044	NA
X^-	20	2.5	0.08	0.32
X^+	35	7.0	0.05	0.093

Supplementary Table 6. Comparison of biexciton Auger rates calculated by different approaches for QDs with the first-exciton absorption peak of CdSe core at 630 nm and 4 or 8 monolayers of shell. Auger rates of negative and positive trions are determined by the QYs and PL decay dynamics of the dim states of blinking trajectories. With superposition principle, biexciton Auger rates are calculated as $k_{Auger,XX}$. Alternatively, the biexciton Auger rates ($k_{Auger,XX'}$) are also calculated with the QY and PL decay dynamics of biexciton via micro-liquid film approach. The average values and standard deviations for 10 individual QDs are presented for the first approach.

Sample	Auger rate of negative trion, $k_{Auger,\ X-}$ (ns^{-1})	Auger rate of positive trion, $k_{Auger,X+}$ (ns^{-1})	Auger rate of biexciton from superposition principle, $k_{Auger,XX}$ (ns^{-1})	Auger rate of biexciton via micro-liquid film, $k_{Auger,XX'}$ (ns^{-1})
**$CdSe_{630}$/4CdS**	0.34$\pm$0.05	0.08$\pm$0.02	0.84$\pm$0.14	0.95
**$CdSe_{630}$/8CdS**	0.10$\pm$0.03	0.18$\pm$0.05	0.55$\pm$0.17	0.57

Supplementary Note 1. Photoluminescence (PL) properties of ensemble CdSe/CdS core/shell QDs.

The zinc-blende CdSe/CdS core/shell QDs with different core sizes and/or different shell thicknesses were synthesized according to the method reported in our previous report[1]. All QD samples used in this work possess nearly unity PL quantum yield, similar PL peak width for ensemble and the corresponding single dots, and mono-exponential PL decay dynamics (Supplementary Table 1, Supplementary Table 2, and Supplementary Figure 2). Near unity PL quantum yield of single-exciton is of critical importance from another viewpoint. At present, the emission quantum yield of a multi-carrier state is measured by comparing with that of the corresponding single exciton state.

As shown in Supplementary Table 1, Supplementary Table 2, and Supplementary Figure2, upon epitaxial growth of the CdS shells onto QDs with a given core size, absorption and PL spectra gradually shift to red and mono-exponential PL decay lifetime increases. In comparison, keeping the same shell thickness but increasing the core size, one would observe significant red-shift of the spectra but small change of the mono-exponential lifetime. Significant red-shift of the spectra suggests that both core and shell dimensions impact quantum confinement. Difference on influence of PL decay lifetime means that spatial distribution of electron and hole wavefunctions of single-exciton is affected differently by the dimensions of the core and shell. This is so because, with near unity PL quantum yield, PL decay lifetime is determined by the electron and hole envelop wavefunction overlapping and the corresponding transition dipole of single-exciton.

Supplementary Note 2. Calculation of Auger recombination rates of biexciton and trions.

As the PL QY of single-exciton is near unity (Figure 1b and Supplementary Note 1), non-radiative recombination of single-exciton is negligible and the mono-exponential

decay rate measured should be the radiative recombination rate of single-exciton ($k_{rad,X}$). Furthermore, for a multi-carrier state, Auger recombination is the only non-radiative decay pathway and its rate can be determined as $k_{Auger} = (1 - QY) \times k$ by measuring its emission QY and rate (k).

For biexciton, with biexciton QY (QY_{XX}) and decay rate ($k_{XX} = 1/\tau_{XX}$, τ_{XX} being the measured biexciton decay lifetime), the radiative and Auger non-radiative recombination rates ($k_{rad,XX}$ and $k_{Auger,XX}$) can be calculated as $k_{rad,XX} = QY_{XX} \times k_{XX}$ and $k_{Auger,XX} = (1 - QY_{XX}) \times k_{XX}$, respectively. The same for trion, with QY_{X^*} and k_{X^*}, the radiative and Auger non-radiative recombination rates ($k_{rad,X^*}$and k_{Auger,X^*}) can be calculated as $k_{rad,X^*} = QY_{X^*} \times k_{X^*}$ and $k_{Auger,X^*} = (1 - QY_{X^*}) \times k_{X^*}$.

Supplementary Note 3. Optical properties of QD micro-liquid film.

As described in the main text, the biexciton properties of QDs can be accurately measured with micro-liquid film approach. The micro-liquid film of QDs is made by sandwiching ~10 μm thick QD solution (~60 QD/μm^3) between two pieces of cover glass. An oil-immersion objective with numerical aperture of 1.49 is used to focus the excitation light to the center of the QD micro-liquid film and collect the emission signals for both steady-state and time-resolved measurements. Supplementary Figure 3a and 3b show the PL spectra and decay curves measured by ensemble QD solution and corresponding micro-liquid film under low-power excitation which are practically identical, indicating that the micro-liquid film approach well retains the properties of ensemble. This consistency holds in a range of concentrations (Supplementary Figure 3c and 3d). Meanwhile, Supplementary Figure 3e and 3f shows that the PL saturation curves and the PL decay curves recovers completely after continuous high power excitation, confirming that diffusion of QDs in the liquid environment efficiently prevents the QDs

from being constantly illuminated for a long period of time and makes them robust against charging and photo-bleaching under high power excitation, and thus yielding quantitatively reproducible results.

Supplementary Note 4. Model of emission saturation for micro-liquid film approach. Calculation of biexciton QY from the PL saturation curve is according to the methods in ref.2. In detail, the photon number absorbed per dot per excitation pulse is considered as a Poisson distribution $\mathrm{P}(m,\langle N\rangle) = \langle N\rangle^m e^{-\langle N\rangle}/m!$ with an average value $\langle N\rangle$. At a given excitation, the total PL intensity measured from a QD sample can be written as

$$I = \sum_{m=1}^{\infty} P(m,\langle N\rangle) \sum_{m=1}^{N} Q_{mX} \qquad \text{(S1)}$$

where Q_{mX} is the quantum yield of the *m*-exciton state.

Only single-exciton emission is observed at long delay time (e.g. 40-50 ns), when multi-exciton recombination completes. As excitation power increases, the single-exciton emission intensity saturates following the function

$$I_X = \sum_{m=1}^{\infty} P(m,\langle N\rangle) Q_X = Q_X\big(1 - P(0,\langle N\rangle)\big) = Q_X\big(1 - e^{-\langle N\rangle}\big) = Q_X(1 - e^{-C\mu}) \qquad \text{(S2)}$$

where $P(m,\langle N\rangle)$ is a Poisson distribution function; Q_X is the single-exciton quantum yield; C is the scaling factor depending on the absorption cross section; μ is the excitation power. Considering Q_X is unity for high quality QD sample, C and thus the x-axis $\langle N\rangle$ can be determined by fitting the single-exciton PL saturation curve with the function above.

For multi-exciton emission, statistical scaling of radiative and non-radiative decay rates are $k_{r,m} = m^2 k_{r,m}/4$ and $k_{nr,m} = m^2(m-1)k_{nr,2}/4$. Thus $Q_{mX} = k_{r,m}/\big(k_{r,m} + k_{nr,m}\big)$. The total emission intensity saturation curve is essential a function

of Q_{2X} and $\langle N \rangle$. Fitting the total emission saturation curve in Supplementary Figure 6 with the model above, the biexciton quantum yield for $CdSe_{650}$/6CdS QDs was determined as $Q_{2X} = 0.2$.

Supplementary Note 5. Core radius and shell thickness dependence of biexciton radiative rates, Auger recombination rates and quantum yield for CdSe/CdS core/shell QDs.

One can see that the biexciton quantum yield increases with core radius whereas almost independent on shell thickness for CdSe/CdS core/shell QDs. Given single-exciton QY being unity, biexciton quantum yield can be written as

$$QY_{XX} = \frac{1}{1+\frac{k_{Auger,XX}}{k_{rad,XX}}} \tag{S3}$$

where $k_{rad,XX}$ and $k_{Auger,XX}$ are the biexciton radiative recombination rate and nonradiative Auger recombination rate respectively. We found that the core radius dependent biexciton quantum yield is due to a faster decline of $k_{Auger,XX}$ comparing to $k_{rad,XX}$ with core radius increases. With the shell thickness increases, the $k_{Auger,XX}$ and $k_{rad,XX}$ decline in the same pace and thus the biexciton quantum yield keeps constant.

Supplementary Note 6. Rates of recombination channels for exciton and negative trion states for CdSe/CdS core/shell QDs measured with single-dot spectroscopy.

The Auger recombination rates of negative trion determined with single-dot spectroscopy is almost identical to the values of the negatively doped QDs by photochemical doping (Fig. 2f). These results are identical evidence for the most prone to occur trion state in single QD is a negative trion state. In addition, radiative recombination rates should be different for QDs within different local environments due to the local field effect[3] (the

first column in Supplementary Table 3 and the first column in Fig. 2f).

Supplementary Note 7. Calculation of Auger recombination rate of positive trion.

The Auger recombination rate of negative trion (deciphering of charge signs is described below) can be determined by the dim state intensity related to the bright state and the PL decay dynamics of the dim state. The Auger recombination rate of positive trion thus can be calculated by the superposition law[4] that $k_{Auger,XX} = 2(k_{Auger,X^-} + k_{Auger,X^+})$.

We investigated the difference between the $k_{Auger,XX}$ values, and thus k_{Auger,X^+} values, calculated by the biexciton QYs and lifetime determined by the micro-liquid film approach and single-dot spectroscopic approach for a QD. The results of k_{Auger,X^+} values obtained via the two methods are almost identical as shown in Supplementary Figure 10.

Supplementary Note 8. Deciphering the charge sign and charge number of the dim states

While changing the surrounding atmosphere from air to oxygen, the discharging rate was highly increased and the dim state was almost eliminated[5]. This result implies that the dim state is a negatively charged state that can readily be oxidized (as shown in Supplementary Figure 13).

As demonstrated by previous work[6], the charge sign of a dim state can be determined by combining PL blinking trace, PL decay dynamics and $g^{(2)}$ trace of the state. In detail,

for a high quality QD sample, the PL QY of a dim state can be calculated from the blinking trace considering the bright state has unity PL QY and nonradiative recombination is negligible. Combining with PL decay dynamics, radiative recombination rate (k_{rad,X^*}) and nonradiative Auger recombination rate (k_{Auger,X^*}) can be figured out (as shown in Supplementary Table 4 and Supplementary Table 5). The PL QY ratio of a charged biexciton to the corresponding charged single exciton can be determined by a second-order photon intensity correlation measurement as shown in Supplementary Figure 14b, with $g_0^{(2)} = QY_{XX^*}/QY_{X^*}$.

Considering the radiative and nonradiative Auger recombination pathways, the PL QY ratios of biexciton to single exciton for negatively charged and positively charged states respectively read

$$\frac{QY_{XX^-}}{QY_{X^-}} = \frac{4k_{rad,X}+2k_{nr,X}+2k_{Auger,X^-}}{4k_{rad,X}+k_{nr,X}+6k_{Auger,X^-}+3k_{Auger,X^+}} \quad \text{(S4)}$$

$$\frac{QY_{XX^+}}{QY_{X^+}} = \frac{6k_{rad,X}+3k_{nr,X}+3k_{Auger,X^+}}{6k_{rad,X}+k_{nr,X}+6k_{Auger,X^+}+3k_{Auger,X^-}} \quad \text{(S5)}$$

where $k_{nr,X}$ is the intrinsic nonradiative decay rate which is negligible for high quality QDs. From Eq. S4 and S5, one notes that the sign of charge has a direct influence on QY_{XX^*}/QY_{X^*} which can be used to identify the sign.

Take $CdSe_{630}$/4CdS as an example, we firstly assume that the dim state with 20% PL QY ('Dim 1' in Figure 4 in the main text) is a negative trion emission state, that is k_{Auger,X^-} = 0.32 ns^{-1}, k_{Auger,X^+} = 0.093 ns^{-1} and $k_{rad,X}$ = 0.044 ns^{-1} as shown in Supplementary Table 5. Thus $\frac{QY_{XX^-}}{QY_{X^-}}$ is calculated as 34.4% which highly agrees with the $g_0^{(2)}$ value of 35% measured using second-order photon intensity correlation experiment (see Supplementary Figure 14). These results confirm that the dim state with 20% QY is a negatively charged state for $CdSe_{630}$/4CdS.

To further confirmation, we also do the similar calculation with the assumption of the dim state with 20% QY being a positively charged state. In this case with k_{Auger,X^-} = 0.093 ns^{-1}, k_{Auger,X^+} = 0.32 ns^{-1} and $k_{rad,X}$ = 0.044 ns^{-1}, $\frac{QY_{XX^+}}{QY_{X^+}}$ is 49.7% which is much higher than the $g_0^{(2)}$ value measured for the state. Therefore the dim state with 20% QY for $CdSe_{630}$/4CdS should not be a positively charged state.

However second-order photon intensity correlation measurement is hard to perform on 'Dim 2' for its rare occurrence. As the dim state with lower QY for $CdSe_{630}$/4CdS is deciphered as a negatively charged state, the dim state with higher QY can only be a positively charged state. This is because a double-charged negative state would have a QY value even lower than the single-charged negative state.

Similar to above, 'Dim 1' for $CdSe_{630}$/8CdS is also deciphered as a negatively charged state which is in agreement with literatures[6-9]. *Dim 2* with lower QY than 'Dim 1' could be either a positively charged state (X^+) or a double-charged negative state (X^{2-}). By counting the radiative recombination pathways for X^-, X^+ and X^{2-} states, the relation between the rates of the radiative recombination rates of the states are $k_{rad,X^-} = k_{rad,X^+}$ and $k_{rad,X^-} = \frac{2}{3} k_{rad,X^{2-}}$. With the almost identical radiative rates of the two dim states (being 0.069 ns^{-1} and 0.072 ns^{-1} respectively) as shown in Supplementary Table 4, the dim state with lower QY ('Dim 2') for $CdSe_{630}$/8CdS is deciphered as a positively charged state instead of a double-charged negative state.

Supplementary Note 9. Computational methods for Auger rate calculation.

The Auger rate is calculated by the phenomenological formula under the standard time-dependent perturbation theory[4]:

$$W_i = \frac{\Gamma}{\hbar}\sum_n \frac{|\langle i|\Delta H|f_n\rangle|^2}{(E_{f_n}-E_i)^2+(\Gamma/2)^2} \tag{S6}$$

where $|i\rangle$ and $|f_n\rangle$ are the initial and final Auger electronic states, E_i and E_{f_n} are their eigenenergies, and ΔH is the Coulomb interaction. Γ is a broadening parameter that accounts for the finite lifetime of the Auger final states due to electron-phonon coupling. By using a single Slater determinant to represent $|i\rangle$ and $|f_n\rangle$, the Auger rate for negative trion (W_i^e) and positive trion (W_i^h) can be calculated by:

$$W_i^e = \frac{\Gamma}{\hbar}\sum_n \frac{|\boldsymbol{J}(e^1,e^2,e_n,h)-\boldsymbol{J}(e^2,e^1,e_n,h)|^2}{(E_{\text{gap}}-E_{e_n}+E_{\text{CBM}})^2+(\Gamma/2)^2} \tag{S7}$$

$$W_i^e = \frac{\Gamma}{\hbar}\sum_n \frac{|\boldsymbol{J}(h^1,h^2,h_n,e)-\boldsymbol{J}(h^2,h^1,h_n,e)|^2}{(E_{\text{gap}}+E_{h_n}-E_{\text{VBM}})^2+(\Gamma/2)^2} \tag{S8}$$

Here e^1, e^2, and h are the two CBM states and one VBM state involved in the negative trion, and e_n is the final state in the higher conduction band. h^1, h^2, and e are the two VBM states and one CBM state involved in the positive trion, and h_n is the final state in the lower valence band. $\boldsymbol{J}(j,k,l,m)$ is the Coulomb integral of the form

$$\boldsymbol{J}(j,k,l,m) = \iint \varphi_j^*(\mathbf{r})\varphi_k^*(\mathbf{r}')\frac{e^2}{\epsilon(\mathbf{r},\mathbf{r}')|\mathbf{r}-\mathbf{r}'|}\varphi_l(\mathbf{r})\varphi_m(\mathbf{r}')d\mathbf{r}d\mathbf{r}' \tag{S9}$$

where $\{\varphi_i\}$ are single particle wavefunctions, and $\epsilon(\mathbf{r},\mathbf{r}')$ is the dielectric function which takes account for the screening effect. To describe the different screening effect inside and outside the QD, we used the regional screening developed in Ref.4:

$$\frac{1}{\epsilon(\mathbf{r},\mathbf{r}')} = 1 + \left(\frac{1}{\epsilon_{\text{in}}(\mathbf{r},\mathbf{r}')} - 1\right) m(r)m(r') \tag{S10}$$

where $m(r)$ is a mask function that changes smoothly from 1, when r is inside the QD, to 0, when r is outside. $\epsilon_{\text{in}}(\mathbf{r},\mathbf{r}')$ is the screening inside the QD, and we have used the proposed model in Ref.10 which includes the G-space dependence.

In our calculation, the surfaces of the constructed QDs are passivated by pseudo H atoms, and the structures are relaxed using the valence force field (VFF) method[11]. The wavefunctions of the QDs are obtained using the charge patching method (CPM) and folded spectrum method (FSM). The motif based CPM produces *ab initio* quality charge

densities for large systems without actually doing self-consistent calculations[12]. The FSM is a linear-in-size method that enables calculation of the eigen solutions of a Schrodinger equation in a desired energy window[13]. The VFF+CPM+FSM approach allows thousand-atom calculations with *ab initio* accuracy. In the calculations, a 30 Ry energy cutoff is used for planewave expansion. The local density approximation for exchange-correlation energy[14] and Troullier-Martins norm-conserving pseudopotentials are adopted[15]. Spin-orbit coupling is included. The Auger rates for negative and positive trions are calculated including final states within an energy window of ~0.2 eV, centered around the energy $Egap+E_{CBM}$ and $E_{VBM}-Egap$, respectively. In practical, this energy window can contain thousands of eigen states. Here 40 eigen states and their wavefunctions around $Egap+E_{CBM}$ and $E_{VBM}-Egap$, were explicitly calculated. We then assumed that all the eigen states in the 0.2 eV window have similar characters as those 40 calculated states, and their energy distribution is estimated based on the calculated DOS in the window. When the initial states are degenerate or nearly degenerate, a configuration-interaction (CI) expansion of the many-body states was used to account for the coupling between the nearly degenerate Slater determinants. The broadening parameter Γ is 10 meV.

Supplementary References

1. Zhou, J. H. *et al.* Ideal CdSe/CdS core/shell nanocrystals enabled by entropic ligands and their core size-, shell thickness-, and ligand-dependent photoluminescence properties. *J. Am. Chem. Soc.* **139,** 16556-16567 (2017).
2. Park, Y. S. *et al.* Near-unity quantum yields of biexciton emission from CdSe/CdS nanocrystals measured using single-particle spectroscopy. *Phys. Rev. Lett.* **106,** 187401 (2011).
3. Zhu, M. Y. *et al.* Effects of local dielectric environment on single-molecule spectroscopy of a CdSe/CdS core/shell quantum dot. *Acs Photonics* **5,** 4139-4146 (2018).
4. Wang, L. W., Califano, M., Zunger, A., Franceschetti, A. Pseudopotential theory of Auger processes in CdSe quantum dots. *Phys. Rev. Lett.* **91,** 056404 (2003).
5. Lorenzon, M. *et al.* Reversed oxygen sensing using colloidal quantum wells towards highly

emissive photoresponsive varnishes. *Nat. Commun.* **6,** 6434 (2015).
6. Xu, W. W. *et al.* Deciphering charging status, absolute quantum efficiency, and absorption cross section of multicarrier states in single colloidal quantum dots. *Nano Lett.* **17,** 7487-7493 (2017).
7. Qin, W., Guyot-Sionnest, P. Evidence for the role of holes in blinking: negative and oxidized CdSe/CdS dots. *ACS Nano* **6,** 9125-9132 (2012).
8. Liu, F. *et al.* Spin dynamics of negatively charged excitons in CdSe/CdS colloidal nanocrystals. *Phys. Rev. B* **88,** 035302 (2013).
9. Javaux, C. *et al.* Thermal activation of non-radiative Auger recombination in charged colloidal nanocrystals. *Nat. Nanotech.* **8,** 206-212 (2013).
10. Franceschetti, A., Fu, H., Wang, L. W., Zunger, A. Many-body pseudopotential theory of excitons in InP and CdSe quantum dots. *Phys. Rev. B* **60,** 1819-1829 (1999).
11. Williamson, A. J., Wang, L. W., Zunger, A. Theoretical interpretation of the experimental electronic structure of lens-shaped self-assembled InAs/GaAs quantum dots. *Phys. Rev. B* **62,** 12963-12977 (2000).
12. Wang, L. W. Charge-density patching method for unconventional semiconductor binary systems. *Phys. Rev. Lett.* **88,** 256402 (2002).
13. Wang, L. W., Zunger, A. Solving schrodingers equation around a desired energy - application to silicon quantum dots. *J. Chem. Phys.* **100,** 2394-2397 (1994).
14. Ceperley, D. M., Alder, B. J. Ground-state of the electron-gas by a stochastic method. *Phys. Rev. Lett.* **45,** 566-569 (1980).
15. Troullier, N., Martins, J. L. A straightforward method for generating soft transferable pseudopotentials. *Solid State Commun.* **74,** 613-616 (1990).
